# Evaluating the Efficacy of Acupotomy Subcision and Hyaluronic Acid Injections for Neck Wrinkles: A Randomized Trial

**DOI:** 10.1111/jocd.70214

**Published:** 2025-04-29

**Authors:** Taolue Ni, Fang Ren, Tianqi Zhang, Dong Hua, Gang Chen, Hao Chen

**Affiliations:** ^1^ Department of Plastic Surgery and Wound Repair Surgery, Anqing Municipal Hospital Anqing China; ^2^ Department of Dermatology, Affiliated Jinling Hospital Medical School of Nanjing University Nanjing China; ^3^ Department of Plastic Surgery, Jiangsu Province Hospital of Chinese Medicine Nanjing China

**Keywords:** acupotomy subcision, hyaluronic acid injections, neck wrinkles, needle knife scripping

## Abstract

**Background:**

Horizontal neck wrinkles are a prevalent sign of aging, with emerging research highlighting the effectiveness of treatments such as acupotomy subcision (AS) and hyaluronic acid (HA) injections in rejuvenating aged skin.

**Aims:**

This study aims to evaluate and compare the efficacy of three distinct treatment modalities for neck wrinkles: AS, HA injections, and a combination of both. The primary objective is to determine which treatment approach offers the most effective and satisfactory outcomes in terms of wrinkle reduction and skin rejuvenation.

**Methods:**

In this randomized clinical trial, 30 female participants with neck Allergan Transverse Neck Lines Scale (ATNLS) scores ranging from 3 to 4 were randomly allocated into three treatment groups: AS, HA, and AS+HA. Each participant received treatment once a month for 3 months, followed by clinical assessments at 1 month and 6 months posttreatment. The assessments included ATNLS scores, Global Aesthetic Improvement Scores (GAIS), and analysis of skin elasticity and collagen levels.

**Results:**

All treatment groups demonstrated improvements in ATNLS scores and GAIS after the final treatment. Notably, the AS+HA group showed a significantly higher number of participants reporting beneficial results at the 6‐month follow‐up. This group also exhibited a statistically significant increase in skin elasticity and collagen levels compared to the other groups.

**Conclusion:**

The combined approach of HA and acupotomy subcision is effective in treating horizontal neck wrinkles, leading to significant improvements in skin elasticity and collagen levels. This study suggests that the synergistic effect of AS and HA may enhance the overall efficacy of treatments for reducing neck wrinkles, providing a promising option for patients seeking noninvasive neck rejuvenation.

## 1 Introduction

The growing demand for cosmetic procedures in areas beyond the face is notable. Patients frequently seek to extend the cosmetic benefits to the neighboring neck region [1, 2]. While horizontal wrinkles on the neck are recognized as a significant characteristic of aging in this area, these static lines may appear at any age; they often become more pronounced as skin loses elasticity over time [3]. An increasing number of individuals are pursuing effective solutions to diminish or eliminate neck wrinkles associated with the aging process.

Present methods for neck rejuvenation encompass laser [4], botulinum toxin [5], filler [6], etc. However, it is difficult for any single treatment to deliver satisfactory results. To achieve the best possible outcomes, it is essential to create personalized treatment plans that take into consideration various individual factors, including the patient's age, the texture of their neck skin, the degree of sagging, and their financial situation. By tailoring these plans to the unique characteristics of each patient, cosmetic doctors can increase the likelihood of successful treatment outcomes. Furthermore, the anatomical structure of the neck complicates the treatment process. The skin in this area is notably thin and is populated with a dense network of blood vessels and nerves, which raises concerns regarding the safety and effectiveness of various treatments [7]. Because of these complexities, further research and investigation are crucial to enhance the overall experience for patients undergoing procedures aimed at reducing neck wrinkles.

Earlier research has indicated that the injection of hyaluronic acid (HA) filler can serve as an efficient approach to diminish or eradicate horizontal neck wrinkles [8]. Appropriate fillers featuring non‐cross‐linked HA, along with amino acids, vitamins, and additional elements, can enhance the comfort of injections, improve penetration effectiveness, and increase adhesion, leading to satisfactory outcomes without the risk of bumps and the blue Tyndall effect, which refers to the blue discoloration of the skin that can occur when HA fillers are injected into the superficial dermis. This effect is particularly noticeable in areas with thin skin, such as the neck [2].

Acupotomy is a contemporary form of acupuncture that entails placing a needle with a flat blade at its end into the body. Acupotomy subcision (AS) is frequently utilized for managing chronic musculoskeletal pain [9], facilitating the release of adhesions in muscle tissue [10], and enhancing the healing of wounds and scars [11].

This study presents a new method for addressing neck wrinkles through the use of HA combined with AS and aims to investigate the efficacy of AS alone, HA injection alone, and AS in combination with HA injection for improving neck wrinkles. Additionally, we examined the safety and effectiveness of these innovative techniques.

## 2 Materials and Methods

### 2.1 Subject Selection

A total of 30 healthy female participants (average age: 37 years, ranging from 29 to 52 years) who had neck wrinkles rated as 3 or 4 on the Allergan Transverse Neck Lines Scale (ATNLS) [12] (see Table 1) and sought treatment to enhance the appearance of their neck wrinkles were recruited between March and May 2023. They were randomly allotted into three treatment groups, each consisting of 10 participants. Participants in Group HA received HA injections, those in Group AS underwent AS, and individuals in Group AS+HA received a combination of AS and HA injections.

**TABLE 1:** Allergan Transverse Neck Lines Scale.

Grade	Description
0 (none)	No transverse neck lines
1 (minimal)	Superficial transverse neck lines
2 (moderate)	Moderate, effaceable transverse neck lines
3 (severe)	Deep, noneffaceable transverse neck lines
4 (extreme)	Noneffaceable transverse neck furrows with redundant skin

**FIGURE 1:**
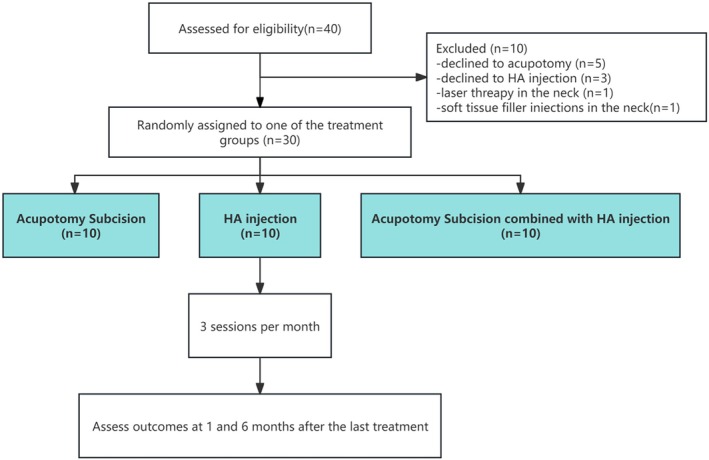
Flowchart showing the procedure for participating and assessment process in this study.

Patients who were pregnant or breastfeeding were not eligible for inclusion in the study, as their specific physiological conditions could affect the outcomes. Furthermore, individuals who expressed a preference against undergoing acupuncture AS or HA treatments were also excluded, ensuring that the study focused on participants who were willing to engage in the proposed interventions. Those with pacemakers were deemed unsuitable for participation due to potential safety concerns associated with certain modalities used in the study. Additionally, individuals suffering from inflammation or malignancies in the neck area were excluded to avoid confounding variables that could compromise the integrity of the study results. Moreover, the study also excluded participants who had received any form of filler injections, laser treatments, ultrasound therapies in the neck region, or botulinum toxin injections targeting the platysma muscle within the 6 months leading up to their enrollment. This exclusion criterion was put in place to minimize the risk of interactions between the effects of previous treatments and the study interventions, thereby ensuring a more accurate evaluation of the outcomes related solely to the treatments being investigated. The flowchart of enrollment is presented in Figure 1.

Each participant indicated their readiness to meet all study conditions and abstain from restricted procedures, such as soft tissue fillers, resurfacing treatments, botulinum toxins, injectable fillers, microdermabrasion, laser and light therapies, and skin tightening during the study period. The research protocol received approval from the Institutional Review Board of NJCUM. All participants provided written informed consent, and a single surgeon carried out all the procedures.

### 2.2 Treatment

To minimize discomfort during the procedure, partial anesthesia in the form of 8% lidocaine cream was applied to the targeted areas. This application occurred 30–60 min in advance of the treatment, allowing sufficient time for the anesthetic to take effect and ensure a more tolerable procedure.

Participants in the AS group were administered AS. Prior to performing AS, every patient received comprehensive physical examinations as well as a thorough photographic assessment. Patients were placed in the supine position to expose the anterior neck and wrinkles. After the asepsis and antisepsis measures were completed, the incision site and insertion areas were injected with local anesthesia (lidocaine, 1% and adrenaline, 1:200 000) for analgesia and hemostasis, respectively. Gently insert a needle knife (0.8 × 50 mm, produced by Suzhou Medical Equipment Factory Co. Ltd) from the point of anesthesia injection into the upper layer of the dermis and perform subcutaneous scripping in the area of the neck wrinkles. The process involves carefully inserting a needle knife into the dermal layer of the skin and performing a series of repeated scripping. This technique effectively creates a space in areas where the epidermal layer and the underlying subcutaneous tissue have become overly adhered to one another. As the scripping progresses, all fibers and adhesion within the subcutaneous space are successfully subcisied; it becomes possible to easily elevate the epidermis. This lifting action brings to light the unique contours and defined shape of the needle knife, demonstrating the effectiveness of the technique. The specific length of each subcutaneous stripping procedure is determined by the length of the neck wrinkles, the length of the needle knife, the curvature of the patients' neck, and the operating habits of the physician.

Participants in the HA group received HA injections. The filler utilized (Hearty1.5 mL, produced by IMEIK Technology Development Co. Ltd.) is a sodium hyaluronate composite solution that is specifically designed for injection purposes. This filler is effective in addressing various skin concerns, particularly those related to the appearance of wrinkles. The targeted treatment area encompassed the horizontal rhytides found in the anterior neck region. To facilitate the procedure, a 30‐gauge, 10‐mm needle was employed, utilizing a retrograde linear threading technique that was applied parallel to the skin within the dermal plane. Each injection administered approximately between 0.05 and 0.1 mL of the filler, which translates to about 10–20 injections per 1 mL of the filler. The punctures were strategically spaced 0.5 cm apart, utilizing an insertion angle of 10°–15° to the skin horizontal surface, with the bevel oriented upwards. Injections were administered into the deep dermis along the horizontal neck wrinkles.

In the AS+HA group, the treatment involved a sequential combination of AS and HA injections. Initially, AS was performed to target the fibrous adhesions beneath the neck wrinkles. A specialized needle knife was inserted into the dermal layer, and subcutaneous scripping was conducted to release the adhered tissues and create a space ready for filler. This step effectively elevated the epidermis, reducing the appearance of wrinkles. Following the AS procedure, HA filler was introduced into the newly created subcutaneous space. The filler was carefully injected using a retrograde linear threading technique, ensuring even distribution along the neck wrinkles. Following each injection session, gentle massage and pressure were applied to the injection sites to distribute the filler into the subcutaneous space stripped before.

The volume of filler required for each patient was determined based on several key factors, including the depth and length of the neck wrinkles, the overall severity of the wrinkles as assessed by ATNLS, and the individual patient's skin elasticity and laxity. These factors collectively influenced the amount of filler needed to achieve optimal aesthetic results and skin rejuvenation. Additionally, our goal for filler administration was to achieve a smooth and wrinkle‐free appearance of the neck, with the treated area appearing flat or slightly elevated after the treatment. When patients tilted their heads down, no wrinkles should be visible at the previously injected sites.

**FIGURE 2:**
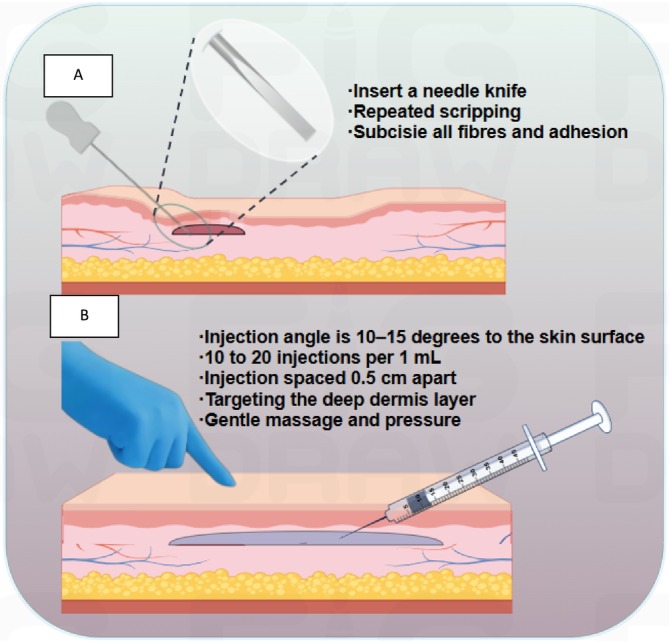
(A) Description of AS. After local anesthesia, a needle knife is inserted and scripping repeatedly until all subcutaneous fibers and adhesions are subcisied. (B) Illustration of AS For the conventional method of HA injection into neck wrinkles, it should be noted that while applying AS in combination, gentle massage is applied to allow the filler to enter the subcutaneous space scripped by the needle knife.(by Figdraw).

Once the combined treatment had been carried out, the area that had been treated was subjected to cooling using ice packs. This step was crucial as it helped to minimize both swelling and edema in the affected region. Additionally, patients were given specific instructions to refrain from engaging in any vigorous neck exercises for a period of seven days following the procedure. This precautionary measure was recommended to facilitate proper healing and to avoid any unnecessary strain on the treated area (Figure 2).

### 2.3 Clinical Assessment

#### 2.3.1 Neck Wrinkle Assessment

Images were taken from both the oblique view and the anterior view during the initial evaluation before any injections occurred and again six months after the completion of the treatment. To maintain the consistency and reliability of the photographs, all subjects were photographed under standardized conditions. Prior to the imaging sessions, the study team confirmed that the neck was devoid of any makeup and that jewelry was removed from the area of treatment. These standardized conditions consisted of employing the same photographer for every picture, keeping camera settings consistent throughout the procedure, ensuring that each participant maintained the same posture in their photographs, and utilizing uniform lighting to achieve a natural and even appearance in all images.

#### 2.3.2 Global Aesthetic Improvement Scale (GAIS)

At 3 months following the treatment, an objective evaluation of the patients' horizontal neck wrinkles was conducted by two plastic surgeons who had no conflicts of interest. These professionals carefully analyzed photographs taken of the patients before and after the treatment, performing their assessments according to the GAIS. This scale categorizes improvement levels from 1 to 5 (5 – very much improved, 4 – much improved, 3 – improved, 2 – no change, and 1 – worse). Furthermore, the patients were requested to provide a subjective assessment of the overall improvement of their horizontal neck wrinkles by GAIS at the 3‐month mark following the treatment.

#### 2.3.3 ATNLS

The scores from the ATNLS were assessed at the beginning of the study and again 3 months after the treatment. The main objective was to determine the percentage of patients who experienced at least a one‐grade improvement.

#### 2.3.4 Analysis of Collagen and Elastin

A CBS skin analysis system (CBS, Wuhan Bose Electronic Co. Ltd., Wuhan, China) was utilized to assess skin elasticity, collagen levels, and oil content in a noninvasive manner. Optical images obtained through microscopy were later converted into negative film. The CBS skin analysis system employs statistical methods based on color gradation recognition and texture scanning within the optical spectrum at 445 nm. Measurements of collagen and elastin levels in the neck skin were conducted at three specific locations using the CBS skin analysis system. These points include: one point at the most obvious point of the wrinkle and the anterior borders in front of the sternocleidomastoid muscle.

## 3 Results

Fourty women underwent eligibility assessment, with 10 failing to meet the inclusion criteria. The remaining 30 women were included in the study, with 10 subjects randomized to the AS group, 10 to the microneedles combined with HA application group, and 10 to the AS+HA group. There were no statistically significant differences in age and BMI among the groups (*p* > 0.05). No subjects were withdrawn from the study after the randomization. The follow‐up period averaged 8.2 months.

### 3.1 GAIS

One month following treatment, participants from the AS, HA, and AS+HA groups indicated responder rates for GAIS of 80%, 90%, and 100%, respectively. Clinicians, on the other hand, noted a 100% GAIS responder rate across all groups. After six months, the responder rates reported by subjects in the AS, HA, and AS+HA groups were 30%, 50%, and 80%, respectively, while clinician‐reported rates were 20%, 40%, and 70%. Notably, the GAIS reported by subjects in the AS+HA group was significantly higher compared to the other two groups(Table 2).

**TABLE 2:** Global aesthetic improvement scale (GAIS) responder rate.

Group	Subject's self‐assessment		Clinician's assessment	
	1 month	6 months	1 month	6 months
AS	80%	30%	100%	20%
HA	90%	50%	100%	40%
AS+HA	100%	80%	100%	70%

### 3.2 ATNLS

**FIGURE 3:**
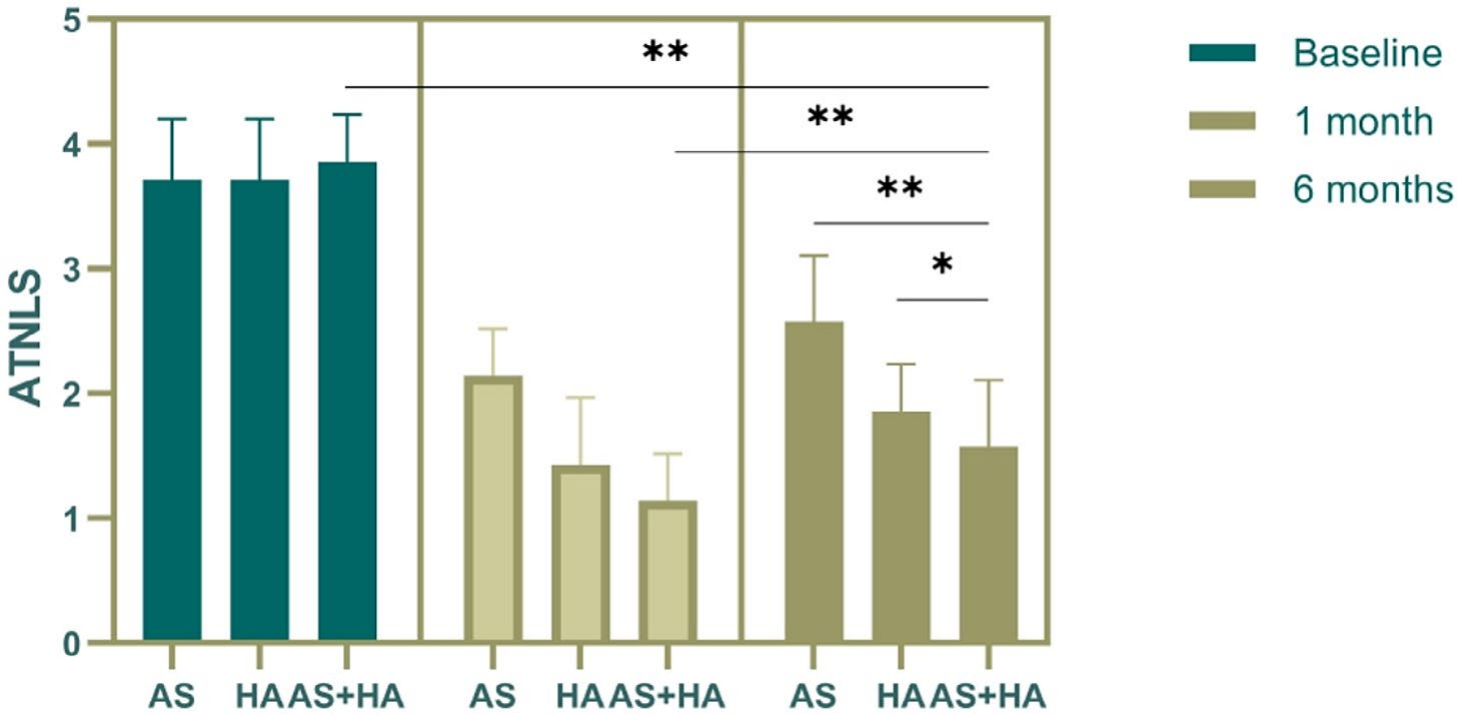
Comparison of ATNLS scores at different time points for the three treatment groups. This figure presents the ATNLS scores for the AS, HA, and AS+HA groups at baseline and at the 1‐month and 6‐month follow‐ups. The scores demonstrate a decrease in neck wrinkle severity over time for all treatment groups, with the AS+HA group showing a statistically significant improvement at the 6‐month mark compared to the other groups. The error bars represent the standard deviation from the mean scores. *p < 0.05, **p < 0.01.

This study found that the ATNLS scores at both the 1‐month and 6‐month follow‐ups were lower than the pretreatment scores in all groups. Additionally, the scores at the 6‐month follow‐up were higher than those at the 1‐month follow‐up. At the 6‐month follow‐up, the ATNLS scores decreased in all three groups compared to baseline, with the AS+HA group showing a statistically significant decrease (Figure 3).

### 3.3 Analysis of Collagen and Elastin

**FIGURE 4:**
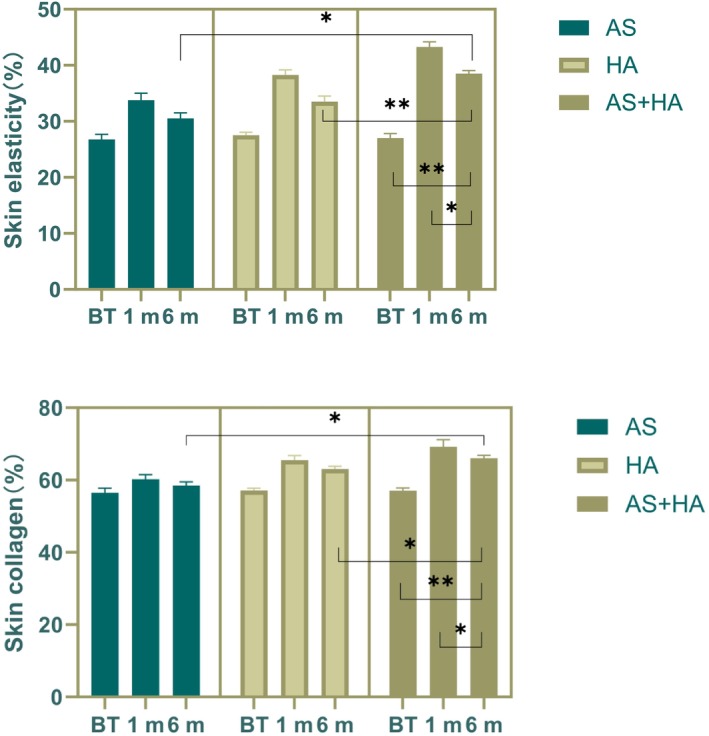
Analysis of skin collagen and elasticity levels in the treatment groups at 1 and 6 months posttreatment. Bar graphs showing the mean collagen levels and skin elasticity measurements obtained from the CBS skin analysis system for the AS, HA, and AS+HA groups at 1 and 6 months posttreatment. The AS+HA group exhibited significantly higher collagen levels and skin elasticity compared to the other groups at the 6‐month follow‐up. BT, before treatment. *p < 0.05, **p < 0.01.

Statistically significant differences were observed in the mean values of skin collagen and elasticity at both 1 and 6 months posttreatment compared to pretreatment in both the AS and HA groups. Furthermore, the AS+HA group demonstrated a significant improvement in skin collagen and elasticity when compared to pretreatment, at 1 month and 6 months posttreatment, as well as the AS and HA groups at 6 months posttreatment (Figure 4).

## 4 Discussion

This study evaluated the clinical efficacy and safety of a comprehensive approach to treat neck wrinkles using HA and AS. The present study provides valuable insights into the treatment of horizontal neck wrinkles through a comparative analysis of AS, HA injections, and their combined application. Our findings underscore the efficacy of the combined approach, AS+HA, in enhancing skin elasticity and collagen levels, leading to a significant reduction in neck wrinkle severity. This synergistic method outperformed individual treatments, demonstrating superior outcomes in terms of wrinkle reduction and skin rejuvenation.

Acupotomy, a contemporary variant of acupuncture, entails the insertion of a needle equipped with a flat knife at its end into the human body [13]. This technique offers dual advantages: It provides the therapeutic benefits of traditional acupuncture alongside a microsurgical effect through the incision. As minimally invasive procedures gain increased attention in today's medical landscape, acupotomy, which merges acupuncture with minimally invasive surgical techniques, has the potential to expand the clinical applications of acupuncture and serve as an effective treatment option for addressing cosmetic issues [14].

Acupotomy subcision stimulation has been shown to significantly promote wound healing, scar recovery, and smooth out wrinkles [15]. This technique of subcision specifically targets the structure of wrinkles, particularly those deeply rooted and anchored by fibrotic strands. By releasing the tension in these areas, subcision allows for the elevation of wrinkles, contributing to a more youthful appearance. The underlying mechanism through which subcision operates involves the severing of fibrous septal bands located within the subcutaneous tissue. This process effectively releases the reticular dermis from its tethering connections, facilitating the deposition of new collagen fibers [16]. As a result, this action promotes an enhanced healing response, which plays a crucial role in improving the overall texture of the skin and achieving a smoother topographical surface. These micro‐injuries activate fibroblasts, which are essential for the stimulation of neocollagenesis and neoelastinogenesis. Notably, this process leads to a thickening of the dermis, which helps to smooth out wrinkles by filling in atrophic areas. Moreover, AS initiates angiogenesis [17], thereby improving blood flow and the delivery of nutrients, both of which are critical for the healing process.

As age, there is a notable decline in the skin's ability to retain water and in the overall volume of the dermal matrix. This phenomenon can be attributed to a decrease in the concentration of HA within the skin, which subsequently increases the likelihood of wrinkle formation, especially neck wrinkles [18]. Consequently, addressing these age‐related changes is imperative for maintaining skin health and appearance. HA fillers have garnered attention as a promising solution for treating neck wrinkles. However, the anatomical features of the neck, characterized by thin skin and sparse subcutaneous fatty tissue, present unique challenges. These characteristics can increase the risk of complications, such as the formation of visible bumps and the occurrence of the blue Tyndall effect. Therefore, selecting fillers with appropriate rheological properties and lower viscosity is crucial to minimize these risks and achieve optimal outcomes.

Moreover, employing non‐cross‐linked HA in treatments can provide immediate smoothing effects on neck wrinkles [19]. The incorporation of L‐carnosine may further enhance the treatment's efficacy, potentially extending its duration by minimizing UV‐related damage and promoting collagen regeneration. By understanding and addressing these factors, practitioners can improve the outcomes of aesthetic procedures aimed at rejuvenating the neck area.

The aging skin's dermal layer experiences a reduction in size, a decline in cell count, a continuous loss of the extracellular matrix, and a shrinking vascular network along with dermal papillae [20]. These prevalent factors lead to the formation of wrinkles, which appear as neck wrinkles. In terms of composition, the dermis primarily consists of three key extracellular matrix components: collagen, elastin, and HA. Throughout the aging process, the previously mentioned components of the extracellular matrix are broken down by metalloproteinases and gradually diminished. Several studies suggest that needle knife stripping may help mitigate the degradation of the extracellular matrix [21], thus offering a therapeutic benefit. Additionally, injecting HA can directly restore the extracellular matrix, providing mechanical support while also inducing local inflammation to stimulate the synthesis of the extracellular matrix [22]. After the needle knife stripping, a specific space forms. Following the injection of HA, gentle pressure can be applied to allow for a more uniform distribution of the injection.

It is worth noting that while we used needles for HA injections in this study, the use of cannulas is another common technique in aesthetic procedures. Cannulas offer certain advantages, such as potentially reduced risk of bruising and the ability to cover larger areas with fewer entry points. However, the choice between needles and cannulas often depends on the specific treatment area, the depth of injection required, and the practitioner's preference [23]. Given the relatively small and precise injection sites required for neck wrinkle treatment, needles were chosen for their precision and control. Future studies could explore whether the use of cannulas would yield similar results in this context.

The findings of this study are consistent with existing evidence that highlights the effectiveness of minimally invasive procedures for neck rejuvenation. The combined treatment not only addresses the aesthetic concerns of neck wrinkles but also enhances the skin's natural healing processes by stimulating collagen production and improving blood flow. This noninvasive approach provides patients with a safer and more comfortable alternative, characterized by minimal downtime and high levels of satisfaction.

While the study is limited by a small sample size and a relatively short follow‐up period, the positive outcomes observed suggest that the combined use of AS and HA injections could become a preferred treatment modality for neck wrinkles. Future research should focus on expanding the sample size, extending the follow‐up duration, and exploring the long‐term stability of the results. Additionally, further investigation into the mechanisms underlying the synergistic effects of AS and HA is warranted to optimize treatment protocols and enhance clinical outcomes.

## 5 Conclusion

The study investigated the effects of three different treatments on horizontal neck wrinkles: the application of AS alone, HA alone, and a combination of both AS and HA. Results indicated that each treatment method led to varying levels of clinical improvement in the appearance of neck wrinkles. Notably, the group that received the combined treatment of AS and HA showed significantly better results compared to the other two groups. This suggests that the synergistic effect of using both AS and HA together may enhance the overall efficacy of the treatment for reducing horizontal neck wrinkles.

## Author Contributions

Taolue Ni and Fang Ren had full access to all of the data in the study and took responsibility for the integrity of the data and the accuracy of the data analysis. Hao Chen and Gang Chen were involved in the study concepts and design. All authors were involved in the acquisition, analysis, and interpretation of data. Dong Hua and Tianqi Zhang finished the analysis and drafted the manuscript. All authors read, critically revised, and approved the manuscript. All the named authors meet the International Committee of Medical Journal Editors (CMJE) criteria for authorship for this article and take responsibility for the integrity of the work as a whole and have given their approval for this version to be published.

## Ethics Statement

All research procedures in this study were performed in accordance with the ethical guidelines of the 1975 Declaration of Helsinki. Written informed consent was obtained from all patients.

## Conflicts of Interest

The authors declare no conflicts of interest.

## Data Availability

The data that support the findings of this study are available from the corresponding author upon reasonable request.
